# Dose reduction to normal tissues as compared to the gross tumor by using intensity modulated radiotherapy in thoracic malignancies

**DOI:** 10.1186/1748-717X-1-31

**Published:** 2006-08-29

**Authors:** Tejinder Kataria, Sheh Rawat, SN Sinha, C Garg, NK Bhalla, PS Negi

**Affiliations:** 1 Department of Radiation Oncology, Rajiv Gandhi Cancer Institute and Research Center, N. Delhi, India; 2 Department of Medical Physics, Rajiv Gandhi Cancer Institute and Research Center, N. Delhi, India

## Abstract

**Background and purpose:**

Intensity modulated radiotherapy (IMRT) is a powerful tool, which might go a long way in reducing radiation doses to critical structures and thereby reduce long term morbidities.

The purpose of this paper is to evaluate the impact of IMRT in reducing the dose to the critical normal tissues while maintaining the desired dose to the volume of interest for thoracic malignancies.

**Materials and methods:**

During the period January 2002 to March 2004, 12 patients of various sites of malignancies in the thoracic region were treated using physical intensity modulator based IMRT. Plans of these patients treated with IMRT were analyzed using dose volume histograms.

**Results:**

An average dose reduction of the mean values by 73% to the heart, 69% to the right lung and 74% to the left lung, with respect to the GTV could be achieved with IMRT.

The 2 year disease free survival was 59% and 2 year overall survival was 59%. The average number of IMRT fields used was 6.

**Conclusion:**

IMRT with inverse planning enabled us to achieve desired dose distribution, due to its ability to provide sharp dose gradients at the junction of tumor and the adjacent critical organs.

## Background

Curative doses of radiation in many instances may lead to a good disease control but cause radiation induced chronic morbidities such as interstitial capillary injury of the myocardium leading to an increased incidence of coronary artery disease, cardiomyopathy and pulmonary interstitial fibrosis. These toxicities are dose related and different structures have varying tolerance to radiation. The availability of data on tissue tolerances makes it imperative to respect the tolerance of critical structures such as the heart, lungs, esophagus etc. and reduce associated morbidities while improving the quality of life.

In most clinical situations, the radiation oncologist is compromised in prescription to treat to tolerance doses of normal tissues rather than to specific tumoricidal dose. IMRT has the potential to increase this therapeutic ratio. The use of IMRT as against conventional radiation allows one to sculpt the dose around a complex shaped target, and has the potential to deliver a higher biologically effective dose to the target. A number of studies have demonstrated the superiority of the physical dose distribution of IMRT compared to other modalities with application in brain tumors, head and neck cancers and prostate cancer treatments [1,2]. As compared to conventional beams, the complexity of IMRT dose patterns makes the verification of the match between planned and delivered doses considerably more difficult. The accuracy of delivered doses is a critical issue for ongoing quality assurance in an IMRT program. Several different techniques have been described and used for clinical implementation of IMRT. These include the "step and shoot" auto sequence multileaf collimator (MLC), dynamic MLC and the physical intensity modulation. The "step and shoot" auto sequence MLC technique delivers an intensity modulated photon field by irradiating a sequence of static MLC ports. The dynamic MLC technique delivers an intensity modulated photon field by moving the collimator leaves during irradiation [3]. Physical intensity modulators are being used to deliver IMRT since the advent of inverse planning software [4]. Schulz [5] and Chang et al [6] have shown in a comparative study between different techniques of IMRT that the MLC technology requires considerably longer time (100%–400%) to deliver the treatment as compared to PIM based IMRT. They have also found a better target volume dose uniformity with PIMs. Sherouse has elaborated that the solid filters are the gold standard and MLC can be an acceptable compromise [7]. He has described solid milled physical modulators as the technology of choice for implementing fluence modulation for IMRT. PIMs are more reliable as the photons are absorbed the same way every time by the PIM, whereas the initial validation measurement in MLC may vary a week later [7]. Hence a day-to-day quality assurance is required to maintain an MLC based IMRT programme. The resolution of PIM is greater in one of the two dimensions because of the size of the MLC leaves, which is typically either 1 cm or 5 mm. The problem of time invariance arises with moving tissues. In dynamic MLC, if the target moves left while the right segment is treated and weaves right while the left segment is treated, there is a potential of 100% error [7].

We present our initial experience with the designing, implementation and dosimetric aspects of IMRT plans of 12 patients.

## Materials and methods

It is a heterogeneous population with post chemotherapy Hodgkin's and non Hodgkin's lymphoma, bronchogenic carcinoma, post operative case of soft tissue sarcoma and tracheo bronchial recurrence in a treated case of carcinoma esophagus.

Planning-A thermoplastic cast was made in the treatment position on the simulator using laser beam alignment and fiducial markers were placed on the thermoplastic cast. A planning CT scan with contrast at cross sections of 3 mm was performed after aligning the external fiducial markers to lasers. The CT images were then transferred to the treatment-planning computer through direct cable network.

Contouring of the tumor and critical normal structures was done by the radiation oncologist with the assistance of a radiologist on every CT slice. Prescription of dose to the target and defining dose constraints for the critical normal structure such as the lungs, cord, heart etc. was done keeping in mind the partial tolerances from the published literature [8]. (Table 2) This patient data facilitated virtual reconstruction of patient anatomy with tumor and organs at risk.

**Table 2:** Dose constraints prescribed for organ at risks.

Organ at risk	organ 3/3	Organ 2/3	organ 1/3
Heart	40 Gy	45 Gy	60 Gy
Lung	17.5 Gy	30 Gy	45 Gy
Spinal Cord: Maximum point dose – 45 Gy			

A photon fluence pattern of each individual beam was generated that met the defined dose constraints on the 3 dimensional treatment planning system (3 D TPS – Plato, Nucletron International) with inverse planning and optimization software. The fluence patterns were used to design and cut special Necupur templates on computerized numerically controlled 3 D milling machine (Autimo system, Hek Medizintechnik). These templates were subsequently used to mould physical intensity modulators (PIMs) of Cerro bend [4]. Re simulation was done for verification of isocenter placement as per optimized plan with the help of previously placed fiducial markers. Photon fluence pattern from film dosimetry (Vidar scanner) as well as by portal imaging were matched with that of optimized fluence maps from treatment planning system for each beam.

Percentage PTV receiving 100% prescribed dose (V100), percentage PTV receiving less than 93% dose (V93) and percentage PTV receiving more than 110% of prescribed dose (V110) were evaluated as per Collaborative Working Group (CWG) recommendations [9]. The homogeneity index (H.I.) was calculated by evaluating the percentage variation between 95% and 10% volume of the PTV using the following formula H.I. = D_10_/D_95 _where D_10 _is the dose received by 10% PTV, and D_95 _is the dose received by 95% of the PTV [10,11].

Statistical analysis was done using SPSS software version 10. Disease free survival (DFS) and overall survival (OS) were calculated by Kaplan Meier method. The DFS was calculated from the date of completion of the planned treatment and OS was calculated from the date of commencement of treatment. For calculating DFS, "disease recurrence", "residual disease" and "lost to follow up with disease" were taken as events while for calculating the OS, "cause specific death", "lost to follow up with disease" and "alive with disease" were considered as events.

## Results

The median age was 50 (35–75) years. The median follow up was 15 months. Seven out of twelve patients achieved a complete response (C.R.), two had partial response (P.R.), one had progressive disease, one died of cause other than cancer and one patient was lost to follow up. Of the 2 patients who had P.R., one patient (case 12) underwent salvage chemotherapy and again had only a partial response to second line chemotherapy (3 cycles) and third line chemotherapy (1 Cycle) and was subsequently lost to follow up with disease.

The average number of IMRT fields was 6 (range 5–8).

For PTV, V100 was 76.4% (65%–100%), V93 was 2.9% (0%–10%) and V110 was 9.9% (0%–46%). For GTV, V100 was 76.4% (65%–100%), V93 was 3.08% (0%–10%) and V110 was 7.4% (0%–46%). The homogeneity index (H.I.) calculated by evaluating the percentage variation between 95% and 10% volume of the PTV was 1.1 (1–1.2) and 95% and 10%volume of the GTV was 1.1% (1–1.2) (Table 3). It is important to note that the maximum dose described by the International Commission on Radiation Units and Measurements Report 50 is the region that is encompassed by a 1.5 cm^3 ^[12].

**Table 3:** Evaluation indices.

Case No.	V*100		V*93		V*110		Homogeneity Index		No of IMRT fields
	PTV†	GTV‡	PTV	GTV	PTV	GTV	PTV	GTV	

1	90	90	0	0	2	0	1.2	1.1	5
2	90	90	0	2	30	0	1.2	1.1	6
3	75	75	0	10	5	5	1	1	7
4	70	70	8	2	0	0	1.1	1.1	8
5	66	66	5	2	1	1	1.1	1.1	6
6	100	100	0	0	46	46	1.2	1.1	6
7	66	66	0	0	5	5	1.2	1	6
8	66	66	4	1	2	0	1.1	1.1	6
9	66	66	10	10	15	15	1.2	1.2	7
10	97	97	6	0	10	0	1.1	1.1	6
11	66	66	0	10	3	14	1.2	1.2	6
12	65	65	2	0	0	3	1	1	6
**Average**	**76.4**	**76.4**	**2.9**	**3.08**	**9.9**	**7.41**	**1.1**	**1.1**	**6**

*V 100, V 93, V 110 Percentage volume receiving 100% dose, less than 93% dose and more than 110% dose.

† Planning target volume, ‡ Gross target volume,

With IMRT plans we were able to achieve an average reduction in mean doses by 73% to the entire heart, 69% to two third of the heart, 49% to one third and 69% to the entire right lung, 70% to two third and 54% to one third right lung, 74% to the entire left lung, 61% to two third and 47% to one third left lung with respect to the GTV. (Table 4).

**Table 4:** Normalized total dose to 2 Gray for various structures.

Case no.	GTV*	PTV†	Right lung			Left lung			Heart			Liver		
	Thorax		Whole organ	2/3 rd	1/3 rd	Whole organ	2/3 rd	1/3 rd	Whole organ	2/3 rd	1/3 rd	Whole organ	2/3 rd	1/3 rd

1	43	43	21	28	32	19	27	31	34	16	16	-	-	-
2	57	53	33	28	36	23	22	22	30	25	32	-	-	-
3	59	59	32	23	39	23	30	29	29	23	34	-	-	-
4	59	59	13	15	17	13	28	38	24	15	28	13	13	13
5	50	48	11	11	13	11	12	19	18	12	20	11	12	17
6	60	60	40	34	44	33	29	37	35	30	36	25	22	24
7	53	47	-	-	-	11	19	22	19	13	22	-	-	-
8	62	60	13	16	24	13	14	36	28	14	35	13	13	13
9	-	40	2	4	23	2	10	18	15	9	19	-	-	-
10	45	40	10	13	25	10	20	32	19	17	21	10	10	11
11	-	27	1	3	9	1	10	15	10	5	14	-	-	-
12	40	40	6	12	14	6	6	12	6	12	36	4	5	5

GTV* Gross tumor volume; PTV† Planning target volume.

The mean dose to the whole heart was 20.4 Gy (2 Gy – 35 Gy) and to 1/3^rd ^heart was 21.6 Gy (2 Gy – 39 Gy), 2/3^rd ^of the right and left lungs received a mean dose of 13 Gy (1 Gy – 28 Gy) and 17 Gy (2 Gy – 28 Gy) respectively while the entire right and left lungs received a mean dose of 17.7 Gy (3 Gy – 31 Gy) and 22 Gy (12 Gy – 32 Gy) respectively (Table 4).

### Acute and late toxicities

One patient (case 12) had evidence of asymptomatic patchy bilateral pulmonary opacities as seen on the chest x-ray at 2 months following radiation. She developed symptomatic bilateral pulmonary infiltrates and minimal pleural effusion with fever and breathlessness at rest at 3 months post radiation. The patient was managed conservatively with a short course of antibiotics and tapering steroids and the symptoms subsided by sixth month. Entire right and left lungs received a mean dose of 24 Gy each, 2/3^rd ^right and left lungs received 13 Gy and 20 Gy each and 1/3^rd ^right and left lungs received a dose of 25 and 32 Gy each.

2 year DFS was 59% with a mean of 24.17 months [95% C.I. 13.54, 34.81] and 2 year OS was 59% with a mean of 45.7 months [95% C.I. 26.55, 64.85].

## Discussion

Gross tumor volume (GTV) is taken as the gross extent of the tumor as shown by imaging studies coupled with the findings on physical examination in lymphoma cases and clinical target volume (CTV) was defined at 10 mm from the GTV. In post operative cases, the CTV for every case was individualized according to the drainage areas, information regarding the tumor bed as per surgical notes and knowledge regarding organ motion. The cases of lung carcinoma that were treated with IMRT were inoperable. The concept of gated IMRT is still under evolution and not available at our center. The excursion of the lungs was seen during simulation and due consideration was given to organ motion while contouring the target volumes. The uniformity of margin was not kept if some highly sensitive structure was in the proximity. PTV was placed at 3–5 mm outside the CTV and the beam edge to PTV was placed at 3–4 mm by the medical physicist.

There is paucity of data regarding the practice of IMRT in thoracic malignancies in literature using physical intensity modulators. We have presented the initial observations and results using PIMs and this is the only study highlighting daily reproducibility, accuracy and outcomes using this technique so far available in literature [13]. The only technical advantage of MLC in present time seems to be that it does not involve manufacturing of a physical modulator which is time consuming and that the technologist does not have to go in the treatment room again and again to change the PIM.

Radiation injury to the heart is most often manifested as pericarditis, although other complications such as chronic pericardial effusion or myocardial infarction may occur. There is ample evidence in literature regarding radiation injury from whole heart irradiation for patients with Hodgkin's disease and partial volume radiation induced heart complications from patients treated post operatively for breast cancer [14]. Literature confirms to TD 5/5 of 40 Gy to whole organ or 60 Gy for 1/3^rd ^organ.

In our series, the mean dose to full heart was 14.8 Gy (1 Gy – 35 Gy), two third heart was less than was 15.9 Gy (5 Gy – 30 Gy) and 1/3^rd ^heart received 25.3 Gy (14 Gy – 36 Gy).

The two most important consequences of irradiation to lungs are pneumonitis and pulmonary fibrosis. Pulmonary fibrosis occurs in almost 100% of patients receiving high doses of irradiation [15-17] but may not be of clinical significance if the volume is small enough. This has been reported in a diverse group of patients afflicted with various diseases but mostly from patients with Hodgkin's disease [18-23] and lung cancer [24]. The TD 5/5 for whole lung is 17.50 Gy, 2/3^rd ^lung is 30 Gy and 1/3^rd ^lung is 45 Gy [8].

In our series, 2/3^rd ^of the right and left lungs received a mean dose of 17 Gy (3 Gy – 34 Gy) and 19.4 Gy (10 Gy – 30 Gy) respectively while the entire right and left lungs received a mean dose of 16.5 Gy (1 Gy – 40 Gy) and 13.8 Gy (1 Gy – 33 Gy) respectively (Table 4).

However, there are some areas of concern in planning and delivery of IMRT. Although parameters such as organ movements and daily patient set up variation are accounted for to some extent in the concept of PTV, there is no provision for the shrinkage of the gross tumor and subsequent change in geometry over the course of radiotherapy.

In view of the fact that IMRT introduces steep gradients near the perimeter of both the target volume and normal structures, IMRT can be "less forgiving" than conventional radiation in regard to the effects resulting from such geometric uncertainties.

## Conclusion

A reduced volume of normal tissues receiving radiation should hypothetically decrease the radiation morbidity, permitting escalation of tumor dose, thereby yielding higher rates of tumor control. In our series, it was possible to achieve an average reduction in the mean dose by 73% to the heart, 69% to the right lungs, 74% to the left lungs and 66% to the cord with respect to the GTV. Only one patient (case 10) developed symptomatic pulmonary pneumopathy which was managed conservatively. It was also possible to re-irradiate a thoracic esophageal recurrence with good clinical response in respiratory obstruction.

IMRT is a tool that has already proven its efficacy in head and neck cancers. With the advent of image guided radiotherapy, reduction in planning target volume is envisaged in future. However, we were able to deliver tumoricidal doses to the target in our heterogeneous group of patients without exceeding the tolerance limits of critical target tissues in the vicinity. This may not have been possible if conventional radiation was planned for these patients.

IMRT will open up new vistas in cases of re irradiation wherein critical structures have already received near tolerance doses of radiation.

**Table 1:** Patient characteristics.

**Case No.**	**Age**	**Sex**	**Primary site**
1	54	M	Hodgkin's lymphoma II A-mediastinum
2	64	M	Soft Tissue Sarcoma (PO)*-right chest wall
3	75	M	Non Small cell lung cancer T2N2M0-right upper lobe
4	69	M	Non Small cell lung cancer Stage III-left upper lobe with chest wall infiltration
5	37	F	Soft Tissue Sarcoma (PO)-dome of diaphragm
6	64	M	Non Small cell lung cancer T2N2M0-right upper lobe
7	38	M	Esophagus with Tracheobronchial recurrence
8	75	M	Non Small cell lung cancer Stage III-left upper lobe
9	45	M	Hodgkin's lymphoma III B-mediastinum
10	44	F	Non Hodgkin's lymphoma II A-mediastinum
11	42	M	Non Hodgkin's lymphoma III-mediastinum
12	35	F	Hodgkin's lymphoma II B-mediastinum

* Post operative

**Table 5:** Patient Outcomes

**Case No.**	**Follow up (Months)**	**Disease Status**	DFS* (months)	**OS† (months)**
1	37	Complete Response	30	37
2	15	Complete Response	14	15
3	9	Died (other cause)	4	9
4	0	Status Unknown	0	0
5	6	Complete Response	4	6
6	10	Complete Response	5	10
7	19	Progressive Disease	0	19
8	6	Partial Response	4	6
9	38	Complete Response	30	38
10	27	Complete Response	22	27
11	59	Complete Response	26	59
12	7	Partial Response	7	7

* Disease free survival, † Overall survival
